# Glycerophospholipid Supplementation as a Potential Intervention for Supporting Cerebral Structure in Older Adults

**DOI:** 10.3389/fnagi.2018.00049

**Published:** 2018-03-07

**Authors:** Jeffery M. Reddan, David J. White, Helen Macpherson, Andrew Scholey, Andrew Pipingas

**Affiliations:** ^1^Centre for Human Psychopharmacology, Swinburne University of Technology, Melbourne, VIC, Australia; ^2^Institute for Physical Activity and Nutrition, Deakin University, Melbourne, VIC, Australia

**Keywords:** glycerophospholipid, supplementation, intervention, cerebral structure, older adults

## Abstract

Modifying nutritional intake through supplementation may be efficacious for altering the trajectory of cerebral structural decline evident with increasing age. To date, there have been a number of clinical trials in older adults whereby chronic supplementation with B vitamins, omega-3 fatty acids, or resveratrol, has been observed to either slow the rate of decline or repair cerebral tissue. There is also some evidence from animal studies indicating that supplementation with glycerophospholipids (GPL) may benefit cerebral structure, though these effects have not yet been investigated in adult humans. Despite this paucity of research, there are a number of factors predicting poorer cerebral structure in older humans, which GPL supplementation appears to beneficially modify or protect against. These include elevated concentrations of homocysteine, unbalanced activity of reactive oxygen species both increasing the risk of oxidative stress, increased concentrations of pro-inflammatory messengers, as well as poorer cardio- and cerebrovascular function. As such, it is hypothesized that GPL supplementation will support cerebral structure in older adults. These cerebral effects may influence cognitive function. The current review aims to provide a theoretical basis for future clinical trials investigating the effects of GPL supplementation on cerebral structural integrity in older adults.

## Introduction

Cerebral structure has been observed to decline with increasing age. Age-related reductions in cerebral structural integrity are evident at both the macro- and microstructural levels (e.g., reduced whole and regional volumes, elevated cortical thinning, increased severity of white matter lesions, and decreased integrity of microscopic white matter pathways), and may begin as early as young adulthood (Resnick et al., 2003; Allen et al., 2005; Fotenos et al., 2005; Walhovd et al., 2005; Bendlin et al., 2010; Hsu et al., 2010; Westlye et al., 2010; Sala et al., 2012; Taki et al., 2013a). There is also a growing literature purporting reduced cerebral structural integrity as a significant predictor of cognitive functioning apparent in older age (Davis et al., 2009; Bendlin et al., 2010; Lockhart et al., 2012; Arvanitakis et al., 2016).

Although all adults demonstrate at least some deterioration in cerebral structure (and cognitive function) with age, the trajectory of decline is not fixed. While some adults appear to demonstrate cerebral decline consistent with “normal aging,” others have conditions such as (in order of worsening dysfunction) “age-associated memory impairment” (AAMI), “mild cognitive impairment” (MCI), and Alzheimer's dementia (AD), all of which are characterized by more severe degradation of cerebral structure, as well as cognitive impairment (Anstey and Maller, 2003; Hänggi et al., 2011; Smith et al., 2011; Bosch et al., 2012; Maillard et al., 2012; Wang et al., 2014; Zheng et al., 2014). These conditions are not inevitable features of increasing age, and it may be possible to reduce their incidence within the population though administration of interventions capable of supporting cerebral structure in older adults.

One factor believed to influence cerebral structural integrity with age is nutritional intake (Scholey, 2018). Previous cross-sectional and longitudinal work has indicated that greater ingestion and bioavailability of B vitamins such as B_6_, B_12_, and folate (Erickson et al., 2008; Vogiatzoglou et al., 2008; Tangney et al., 2011; Hsu et al., 2015; Hooshmand et al., 2016; Köbe et al., 2016) or omega-3 polyunsaturated fatty acids (ω3-PUFA; Samieri et al., 2012; Tan et al., 2012; Titova et al., 2013; Pottala et al., 2014; Gu et al., 2016) predicts greater cerebral macro- and micro-structural integrity in older adults. Moreover, data from available clinical trials indicates that chronic supplementation with these nutrients, but also others such as resveratrol, may either enhance cerebral structural integrity, or improve the trajectory of cerebral decline over time (Smith et al., 2010; Douaud et al., 2013; Witte et al., 2013; Jerneren et al., 2015; Köbe et al., 2017; Zhang et al., 2017). It is possible that these structural effects, at least partly, underpin the benefits to cognitive performance in older adults following supplementation (Durga et al., 2007; Yurko-Mauro et al., 2010; de Jager et al., 2012; Witte et al., 2013).

The consumption of phospholipids (PL) and in particular the glycerophospholipids (GPL), may also benefit cerebral structure and subsequently cognitive function in older adults. The GPL species phosphatidylcholine (PC), phosphatidylethanolamine (PE), and phosphatidylserine (PS) are abundant in mammalian cell membranes, and there is growing evidence that provision of these GPL (particularly PC and PS) can improve cognitive function in animals via oral supplementation (Zanotti et al., 1989; Furushiro et al., 1997; Lim and Suzuki, 2000; Suzuki et al., 2001; Kataoka-kato et al., 2005; Yaguchi et al., 2009, 2010; Lee et al., 2010; Babenko and Semenova, 2011; Nagata et al., 2011; Park et al., 2013; Zhang et al., 2015; Qu et al., 2016; Wen et al., 2016a,b) or intraperitoneal/intracerebral injection (Drago et al., 1981; Zanotti et al., 1984; Corwin et al., 1985; Sakai et al., 1996; Blokland et al., 1999; Claro et al., 1999, 2006; Suzuki et al., 2000). Similar results are also evident following oral supplementation in older humans with varying levels of cognitive function (i.e., normal cognitive function with subjective memory complaints, age-related “cognitive dysfunction,” AAMI, MCI, or dementia). The details and findings of these trials are summarized in Tables 1–3.

**Table 1:** GPL supplementation and cognitive function in older adults with subjective memory complaints (trials listed in reverse chronological order) and by subject type.

**Trial**	**Design**	**Subjects**	***N***	**Age (years)**	**Treatment**	**Time**	**Outcomes**
More et al., 2014 (sub-study 1)	R, DB, PC, PG	SMC	72	60–80	SB-PS (300 mg/d) + PA (240 mg/d)	3 mo	No treatment effects on WMS performance when comparing baseline low performers. Significant differences in WMS performance between groups favoring treatment in baseline higher performers
Vakhapova et al., 2014	OLE	SMC	122	72.4 ± 8.3 (naïve); 72.1 ± 7.9 (continuers)	100 mg PS/d (as well as 26 mg DHA + EPA)	15 w	Significantly improved sustained attention and memory recognition performance in the PS-DHA naïve participants (who did not receive PS in the earlier clinical trial)
Richter et al., 2013	OL	SMC	26	50–90 (74.6 ± 1.7)	300 mg/d SB-PS	12 w	Significantly improved memory performance, executive functioning and mental flexibility
Kato-Kataoka et al., 2010	R, DB, PC, PG	SMC	73	T: 59.6 ± 1.0 (high dose)T: 59.1 ± 1.1 (low dose)P: 59.6 ± 1.1	100mg/d SB-PS or 300 mg/d SB-PS	6 mo	Memory performance significantly increased from baseline for all groups. Improved performance on HDS-R test in high dose treatment participants who has poor performance at baseline vs. placebo. Improved delayed verbal memory recall in participant receiving high or low dose treatments vs. placebo
Richter et al., 2010	OL	SMC	8	69.3 ± 3.2	300 mg PS/d (as well as 37.5 mg DHA + EPA)	6 w	Significantly improved delayed verbal recall following treatment
Vakhapova et al., 2010	R, DB, PC, PG	SMC	157	T: 72.9 ± 8.20P: 73.01 ± 8.28	300 mg/d PS (as well as 79 mg DHA + EPA)	15 w	Significantly improved immediate verbal recall. A trend toward reduced time for completing RCF was observed but failed to reach significance (*p* = 0.079)
Jorissen et al., 2001	R, DB, PC, PG	AAMI	120	T: 65.8 ± 1.1 (high dose)T: 65.3 ± 0.9 (low dose)P: 64.6 ± 0.9	300 mg/d SB-PS; 600 mg/d SB-PS	12 w (+3 w placebo washout)	No treatment effects were observed for any measure of cognitive function after treatment or washout

*Design—R, Randomized; DB, Double Blind; PC, Placebo Controlled; PG, Parallel Groups; OL, Open Label; OLE, Open Label Extension; Subjects-SMC, Subjective Memory Complainers; AAMI, Age-Associated Memory Impairment; Age—T, Treatment Group; P, Placebo Group; Treatment—SB, Soybean; PB, Plant Based; BC, Bovine Cortex; PS, Phosphatidylserine; PA, Phosphatidic Acid; DHA, Docosahexaenoic Acid; EPA, Eicosapentaenoic Acid; Time—d, Day; w, Weeks; mo, Months; Outcomes—WMS, Weschler Memory Scale; HDS-R, Hasegawa's Dementia Scare Revised; RCF, Rey Complex Figure*.

**Table 2:** GPL supplementation and cognitive function in older adults with age-related “cognitive deterioration” (trials listed in reverse chronological order).

**Trial**	**Design**	**Subjects**	**N**	**Age (years)**	**Treatment**	**Time**	**Outcomes**
Nagata et al., 2011	OL, PG	CD	310	59–95 (76 ± 1.2)	100 mg/d DLPhtCho; 90 mg/d POPhtCho; Combined 50 mg/d DLPhtCho + 45 mg/d POPhtCho	5 mo	MMSE scores were markedly increased following all treatments. MMSE score increase was significantly greater following dual supplementation compared to single supplementation.
Cenacchi et al., 1993	R, DB, PC, PG	CD	494	T: 77.8 ± 5.6 P: 77.3 ± 6.3	300 mg/d BC-PS	6 mo	Significantly improved verbal memory performance following 3 and 6 months treatment compared to placebo.
Allegro et al., 1987	OL	CD	(30)	72.4 ± 4.8	300 mg PS	60 d	Significantly improved verbal and working memory performance over 60 day's treatment. Increased memory function compared to baseline 30 days post-treatment, though most scores reduced to below 60-day treatment scores.
Caffarra and Santamaria, 1987	OL	CD	30	69.2 ± 5.6	300 mg PS	60 d	Significantly improved verbal memory (acquisition and recall), immediate semantic memory performance as well as attention/concentration.
Villardita et al., 1987	R, DB, PC, PG	CD	170	55–80 (65.7 ± 7.5)	300 mg/d BC-PS	90 d	Significantly improved attention/vigilance, verbal and working memory performance, as well as immediate and delayed semantic memory compared to placebo.

*Design—R, Randomized; DB, Double Blind; PC, Placebo Controlled; PG, Parallel Groups; OL, Open Label; Subjects—CD, Cognitive deterioration; Age—T, Treatment Group; P, Placebo Group; Treatment—BC, Bovine Cortex; DLPhtCho, 1,2-dilynoleoyl-sn-glycero-3-phosphocholine; POPhtCho, 1-palmitoyl-2-oleoyl-sn-glycero-3-phosphocholine; PS, Phosphatidylserine; Time—d, Days; mo, Months*.

**Table 3:** GPL supplementation and cognitive function in older adults with dementia (trials listed in reverse chronological order) and by subject type.

**Trial**	**Design**	**Subjects**	**N**	**Age (years)**	**Treatment**	**Time**	**Outcomes**
Zhang et al., 2015	R, PC, PG	AD	57	T: 74.9 ± 18.2P: 75.3 ± 11.8	300 mg/d PS	20 w	Significantly improved memory performance (vocabulary-picture matching) following treatment
More et al., 2014 (sub-study 2)	DB, R, PC, PG	AD	96	50–90 (75.3)	300 mg/d SB-PS + 240 mg/d PA	2 mo	No further deterioration in ADL score following treatment, though further deterioration evident following placebo. Slight, though non-significant improvements in both groups, though there was a higher proportion of participants in the treatment group progressing from abnormal (≤23) to normal score range (>23) than placebo.
Heiss et al., 1994	R, OL, PG	AD	70	48–79	CT + PS (400 mg/d); CT + P^*^ (1,200 mg/d); CT; SS	6 mo	Significantly improved orientation performance (on MMSE) after 8 and 16 weeks treatment for CT+ PS group relative to SS or CT, but not compared to CT + P^*^. No between group differences on MMSE at 6 months. Within group analysis indicates that no treatment effects were evident for SS or CT. CT+P^*^ demonstrated increased orientation scores at 8 weeks and verbal fluency at 16 weeks. CT+PS demonstrated significantly higher MMSE at 8 and 16 weeks with a trend toward higher scores at 6 months. A similar trend was evident for orientation scores. Visuospatial performance also improved in the CT+PS group.
Heiss et al., 1993	OL, R, PG	AD	80	Not Specified	CT + PS (400 mg/d); CT + P^*^ (1,200 mg/d); CT; SS	6 mo	Significantly improved MMSE score, as well as block span test (short term memory performance) only evident for CP+PS group
Amaducci, 1988	R, DB, PC, PG	AD	142	T: 62.0 ± 7.4P: 62.2 ± 6.9	200 mg/d BC-PS	3 mo	Significantly improved BDRS score following 3 months treatment. Significant improved BDRS score, relative to baseline, was evident 3 months post-treatment.
Yaguchi et al., 2009	OL, PG	MCI, D	67	59–93 (77.1 ± 0.8)	300 mg/d POPhtCho	6 mo	Treatment effects on mean MMSE score. Mean MMSE score was increased following treatment, with no change identified in control subjects.
Granata and Di Michele, 1987	OL	D	35	61–80 (70.94 ± 5.43)	300 mg PS	60 d	Significantly improved verbal and working memory performance
Puca et al., 1987	OL	D	27	55–80 (65.5 ± 8.6)	300 mg PS	60 d	Significantly improved verbal memory performance after 60 days. Some improvements in memory performance were maintained after 30 days no treatment.

Design—R, Randomized; DB, Double Blind; PC, Placebo Controlled; PG, Parallel Groups; OL, Open Label; Subjects—MCI, Mild Cognitive Impairment; AD, Alzheimer's Dementia; D, Dementia; Time—d, Days; w, Weeks; mo, Months; Age—T, Treatment Group; P, Placebo Group; Treatment—SB, Soybean; BC, Bovine Cortex; PS, Phosphatidylserine; PA, Phosphatidic Acid; DLPhtCho, 1,2-dilynoleoyl-sn-glycero-3-phosphocholine; POPhtCho, 1-palmitoyl-2-oleoyl-sn-glycero-3-phosphocholine; CT, Cognitive Training; SS, Social Support; P^*^, Pyritinol; Outcomes—ADL, Activities of Daily Living; MMSE, Mini-Mental State Examination; BDRS, Blessed Dementia Rating Scale.

While the evidence for GPL supplementation benefiting cognitive function is relatively robust, there is comparatively less work investigating their effects on cerebral structure. In a number studies with middle-aged and older rodents, GPL supplementation was found to improve hippocampal cell morphology (Nunzi et al., 1987; Crespo et al., 2004; Qu et al., 2016), as well as elevate cellular proliferation and survival within the dentate gyrus (Maragno et al., 2015). Likewise, 7 months oral supplementation with PE (1,2-dilinoleoyl-*sn*-glycero-3-phosphoethanolamine) has been observed to reduce age-related hippocampal neuron death in senescence accelerated SAMP8 rodents (Yaguchi et al., 2010). However, to date there have been no such studies with older humans.

Interestingly, GPL supplementation may potentially modify a number of factors predicting compromised cerebral structural in older humans. These include elevated homocysteine (HcY) concentrations, unbalanced activity of reactive oxygen species (ROS) activity and increased oxidative stress (OxS), higher levels of pro-inflammatory messengers, as well as poorer cardio- and cerebrovascular functioning. By modifying these factors, GPL supplementation may support cerebral structural integrity, and subsequently cognitive function, in older adults.

In this review, an introduction to GPL—their chemical structure, how they are synthesized *in vivo* and from what foods they are available from—will be presented. Following this, there will be an overview of each of the aforementioned factors, how they relate to cerebral structure (e.g., whole and regional cerebral volume, cortical thinning, severity of white matter lesions, and the integrity of microscopic white matter pathways) as well as cognitive function. This will be complemented with an overview of the available studies investigating the extent to which GPL supplementation modifies, or protects against, these risk factors. Overall, it is anticipated that GPL supplementation, particularly species containing choline and/or ω3-PUFA, may beneficially modify risk factors predicting cerebral structural decline, thereby supporting cerebral structure and subsequently cognitive function in older adults.

## Phospholipids

### What are phospholipids?

The term “phospholipid” (PL) may be used to describe any lipid (fatty acid) with a phosphoric acid residue (Hanahan, 1997). There are two major classes of PL—glycerophospholipids (GPL) and sphingolipids, both being essential components of cellular membranes, including those forming cerebral tissue. This review will focus on GPL, specifically PC, PE, and PS as these are the most common GPL species within mammalian cell membranes (Castro-Gómez et al., 2015), but also the most frequently examined GPL species in relation to neurocognitive health, or risk factors pertaining to neurocognitive health in older adults.

**Figure 1:**
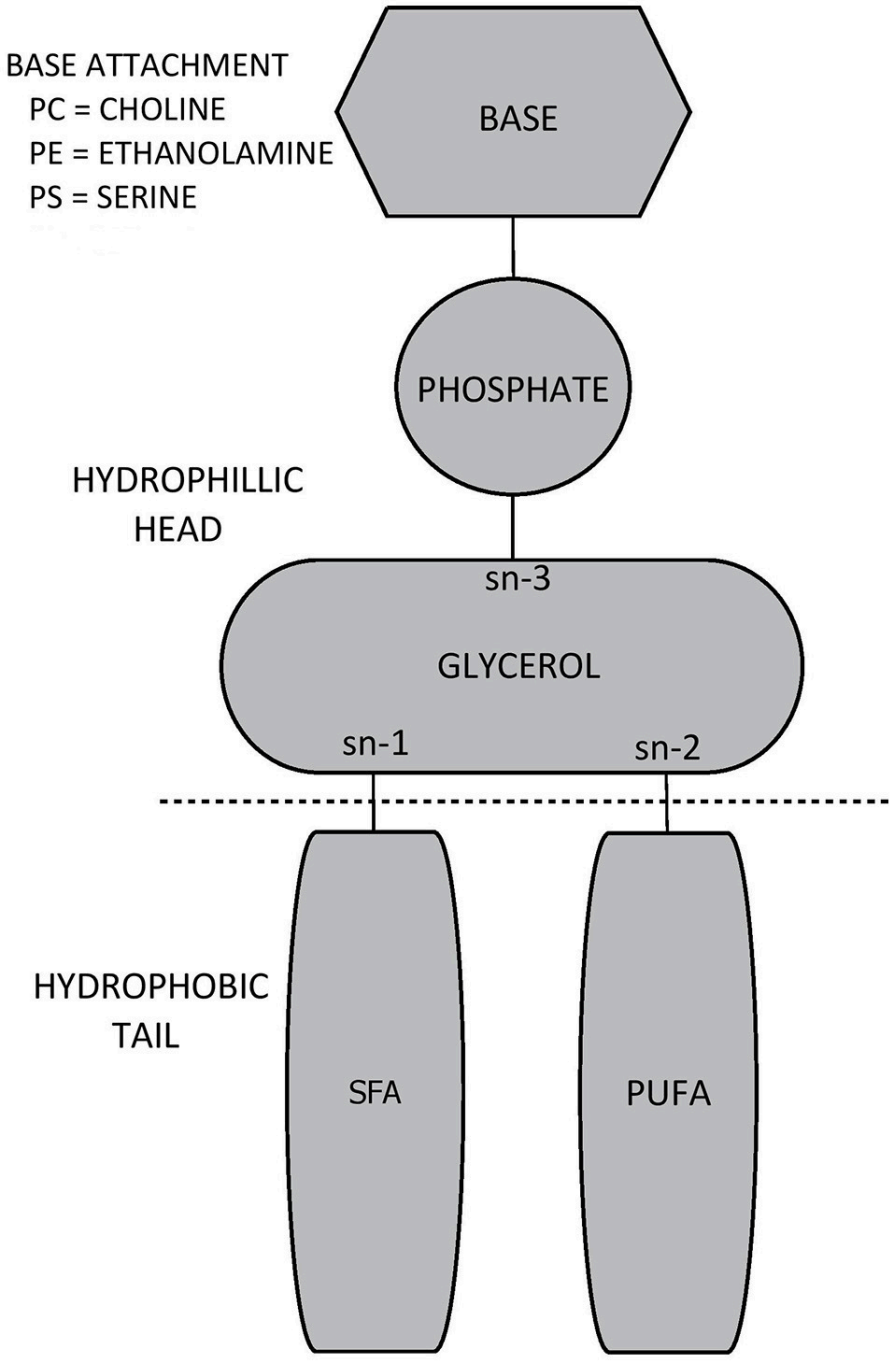
General biochemical structure of a glycerophospholipid.

Glycerol forms the backbone of the GPL molecule and it contains three hydroxyl groups (sn-1, sn-2, and sn-3). A phosphate group distinguishing the overall species of GPL is attached to the glycerol backbone at sn-3 (Ridgway, 2016). The attached phosphate may be choline, ethanolamine, or serine, thereby forming PC, PE, or PS, respectively (Ridgway, 2016). Meanwhile, fatty acids are attached to glycerol at sn-1 and sn-2. Quite often sn-1 is occupied by a saturated fatty acid, while sn-2 is occupied by an unsaturated fatty acid, with polyunsaturated fatty acids being especially prevalent in GPL composing cerebral tissue membranes (Castro-Gómez et al., 2015). The combination of different phosphate groups and fatty acids gives rise to over 1,000 different subspecies of GPL (Vance, 2008). The structure of GPL species evident within mammalian cell membranes (PC, PE, PS as well as phosphatidylinositol) is presented in Figure 1.

The phosphate group at sn-3 forms the hydrophilic head of the GPL molecule, whereas the fatty acids attached at sn-1 and sn-2 form the hydrophobic tail (Castro-Gómez et al., 2015). The presence of a hydrophilic head and hydrophobic tail results in the formation bi-lipid layers when GPL are suspended together in aqueous solutions (Cooper and Hausman, 2007). This proclivity toward forming bi-lipid layers allows GPL (together with sphingolipids and proteins) to form the cell bi-lipid membrane, though different species of GPL appear to be differentially concentrated within membrane layers. PC is primarily located within the outer membrane layer (sometimes termed the outer leaflet) alongside sphingomyelin. Conversely, PE is mostly concentrated within the inner membrane layer (the inner leaflet), with PS being exclusively located within the inner leaflet (Devaux and Zachowski, 1994; Castro-Gómez et al., 2015).

### Biosynthesis of GPL

GPL such as PC, PE, and PS may be synthesized *in vivo* via a number of different pathways. Some of these pathways involve *de novo* synthesis, whereas others involve remodeling of pre-existing GPL.

**Figure 2:**
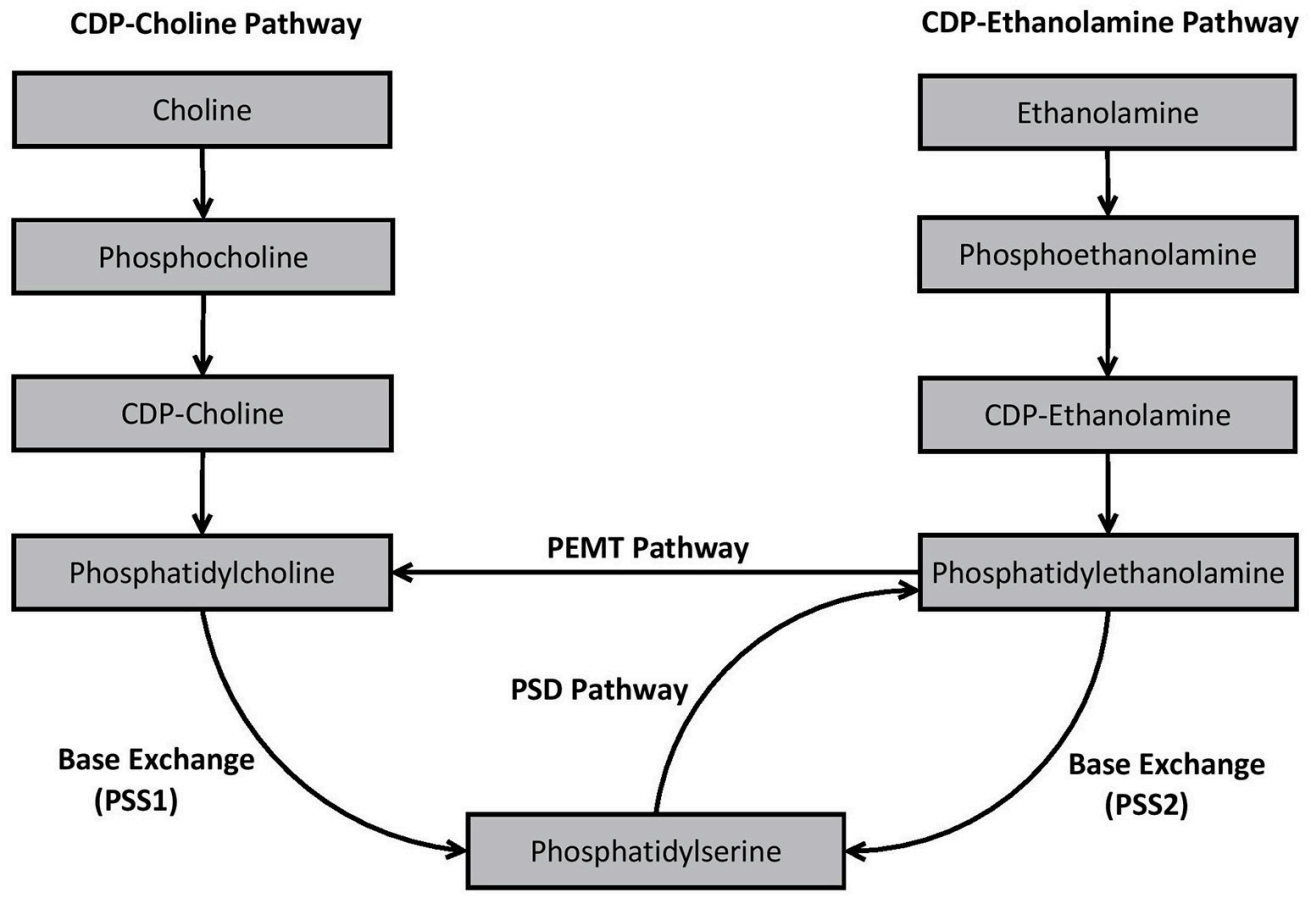
Biosynthesis pathways of phosphatidylcholine, phosphatidylethanolamine, and phosphatidylserine in mammalian cells.

An essential process prior to the *de novo* synthesis of PC and PE (but also phosphatidylinositol) is the initial formation of phosphatidic acid (Kent, 1995). Once synthesized, phosphatidic acid is converted to either 1,2-diacylglycerol or cytidine diphosphate (CDP) diacylglycerol by two different enzymes—phosphatidic acid phosphatase and CDP-diacylglycerol synthase, respectively (Kent, 1995). 1,2-diacylglcerol is important for synthesis of PC and PE, whereas CDP-diacylglycerol is essential for the formation of phosphatidylinositol in mammalian cells (Kent, 1995). In mammalian cells, PS synthesis is not reliant upon phosphatidic acid synthesis. The major *de novo* and remodeling pathways associated with GPL biosynthesis in mammalian cells are outlined in Figure 2.

As shown in Figure 2, PC is produced *in vivo* via two major pathways—the cytidine disphosphocholine (CDP-choline) pathway, and the phosphatidylethanolamine *N*-methyltransferase (PEMT) pathway. The CDP-choline pathway involves *de novo* synthesis of PC whereas the PEMT pathway involves remodeling of pre-existing PE. PE is synthesized analogously to PC via the CDP-ethanolamine pathway, but also by remodeling pre-existing PS via the PS decarboxylase (PSD) pathway. In mammalian cells, PS is primarily synthesized through base exchange reactions mediated by specialized synthases. PS synthase-1 (PSS1) causes free serine to be switched for choline from PC, while PSS2 switches ethanolamine in PE with free serine, thereby formulating PS. Detailed decriptions of the specific biochemical processes involved in each of these pathways have been provided elsewhere (Kent, 1995; Vance, 2008; Vance and Tasseva, 2013).

While different pathways may produce the same general species of GPL (e.g., CDP-choline and PEMT both produce PC), the pathway from which the GPL was synthesized appears to influence the fatty acid composition of the resultant GPL. For example, PC resulting from the CDP-choline pathway typically contains saturated, monounsaturated or diunsaturated fatty acid species, whereas PC synthesized via the PEMT pathway may also contain polyunsaturated fatty acids (PUFA), such as the ω3-PUFA species DHA (Delong et al., 1999; Pynn et al., 2011). Although this may be due to a substrate preference of the PEMT pathway for PE containing ω3-PUFA (Delong et al., 1999), others (e.g., Vance, 2014) suggest that the extent to which DHA enriched PC is produced is more likely dependent upon the availability of DHA within the overall reservoir of PE (Ridgway and Vance, 1988).

Differences in fatty acid composition depending on synthesis pathways are also apparent for PE. PE produced via the CDP-ethanolamine pathway predominately contains mono- or di-unsaturated fatty acid species at sn-2. Conversely, PE derived from the PSD pathway, predominately synthesizes PE containing PUFA such as the ω6-PUFA arachidonic acid (ARA) or ω3-PUFA's such as DHA or eicosapentaenoic acid (EPA) (Bleijerveld et al., 2007). Substrate specificity is apparent during PS synthesis in mammalian cells. Data from several studies indicate that PSS1 and PSS2 preference species of PC or PE containing ω3-PUFA, particularly DHA, compared to other subspecies (Kim et al., 2004; Kimura and Kim, 2013).

### Dietary sources of GPL containing ω3-PUFA

In addition to *in vivo* synthesis, GPL may be obtained from the diet. The estimated dietary intake of GPL is two to eight grams per day, representing one to ten percent of daily fat intake (Cohn et al., 2010). GPL are present in foods such as milk, eggs, skeletal, and organ (e.g., brain) meat from terrestrial animals (e.g., cow or pig), as well as fish (especially krill or squid). GPL may also be obtained by consuming certain seeds and legumes such as rapeseeds, sunflower seeds and soybeans (Weihrauch and Son, 1983).

The types of fatty acids contained within GPL appear to vary between food sources. The GPL derived from foods such as marine fish (e.g., krill or squid), mammalian brain (e.g., cow or pig) or eggs (particularly those from hens whose feed is fortified with ω3-PUFA) appear to contain ω3-PUFA such as DHA (Bourre et al., 1993; Bourre and Dumont, 2002; Favrelière et al., 2003; Chen and Li, 2008). In soybeans, ω3-PUFA is present in the form of α-linolenic acid (ALA) (Bourre and Dumont, 2002; Chen and Li, 2008), which may be later converted to longer chain ω3-PUFA species such as EPA and DHA (Burdge and Calder, 2005). Soybeans, but also fish, mammalian brain and eggs also provide varying levels of ω6-PUFA and other fatty acids (Bourre and Dumont, 2002; Favrelière et al., 2003; Chen and Li, 2008).

Aside from whole foods, GPL may be obtained from commercially available supplements. Importantly, the fatty acid composition of these supplemental GPL will vary depending on the food from which the GPL was extracted. GPL supplements may be consumed as capsules (e.g., krill oil), as well as powdered compounds (summarized in Küllenberg et al., 2012). Supplements in powder form are liquefiable thereby making them easier to consume. This may be particularly important for older adults especially those who experience difficulty swallowing (a common occurrence in the general population termed dysphagia; Aslam and Vaezi, 2013). These adults may be at an increased risk of developing nutritional insufficiency or deficiency (Sura et al., 2012).

Prior work has indicated that the bioavailability of ω3-PUFA may be increased following ingestion of whole foods or supplements containing this species of fatty acids (Popp-Snijders et al., 1986; Bourre et al., 1993; Yaqoob et al., 2000; Bourre and Dumont, 2002; Kew et al., 2004; Ulven et al., 2011; Browning et al., 2012). Other work demonstrates that the bioavailability of PUFA may be enhanced when they are consumed as GPL rather than triglycerides (Wijendran et al., 2002; Ramprasath et al., 2013). As such, GPL supplementation may be an effective method of increasing the bioavailability of nutrients such as ω3-PUFA. Given that other nutrients such as choline may also be attached to GPL, it is likely that GPL supplementation will also boost the bioavailability of these nutrients.

## GPL supplementation as a means of supporting cerebral structure in older adults

### GPL supplementation and factors predicting cerebral structural integrity

The concentrations of PL within cerebral tissue appears to decline with increasing age (Soderberg et al., 1990, 1991; Svennerholm et al., 1994, 1997). As such, increasing the dietary intake of PL, especially GPL, may benefit cerebral structure integrity due their incorporation into neuronal membranes. However, supplementation with GPL may also indirectly support cerebral structure by modifying, or protecting against, a number of factors that appear to predict poorer cerebral structure in older adults. It is these potential indirect effects that form the focus of this review. Specifically, this review will discuss biochemical (HcY, ROS activity and OxS, and inflammation) and biophysical (cardio- and cerebrovascular function) risk factors associated with poorer cerebral structural integrity, but also cognitive decline, and the extent to which GPL supplementation has been observed to modify, or protect against, these factors. There will also be a discussion of the potential pathways through which GPL supplementation may facilitate these effects. Overall, it is anticipated that, GPL supplementation will benefit cerebral structural integrity and subsequently cognitive function in older adults, via modification of, or protection against, the aforementioned factors.

### Homocysteine

#### Homocystiene and cerebral structure in older adults

HcY is a sulfur containing amino acid derived from s-adenosylhomocysteine, a by-product of methyl reactions involving *s-*adenosylmethionine (Selhub et al., 2000). A number of studies with older adults have determined that elevated HcY is a significant predictor of reduced total and regional cerebral tissue volumes (Williams et al., 2002; den Heijer et al., 2003b; Firbank et al., 2010; Narayan et al., 2011a; Rajagopalan et al., 2011; Tangney et al., 2011; Feng et al., 2013; Madsen et al., 2015), increased frequency and volume of white matter lesions or silent brain infarcts (Vermeer et al., 2002; Wright et al., 2005; Seshadri et al., 2008; Raz et al., 2012), as well as reduced cerebral microstructural integrity (Bettcher et al., 2014; Hsu et al., 2015). Given the link between measures of cerebral macro- and microstructural integrity and cognitive functioning, it follows that HcY may also be an important predictor of cognitive function. In fact multiple studies have observed an increased risk of developing MCI and dementia in adults with elevated HcY (Launtenschlager et al., 2005; Kim et al., 2007; Whalley et al., 2014), but also poorer performance across a number of cognitive domains (Narayan et al., 2011b; Allam et al., 2013; Jochemsen et al., 2013; Parizkova et al., 2017).

While HcY concentrations may be influenced by non-modifiable factors such as genetics and age, there are also a number of modifiable factors believed to impact HcY concentrations. One of the most important modifiable factors is nutritional intake, in particular B vitamin ingestion/absorption (Joosten et al., 1996; Jacques et al., 2001; Refsum et al., 2006). Vitamins B_6_, B_12_, and folate are essential for HcY metabolism, with lower intake or bioavailability of these vitamins predicting elevated HcY concentrations (Jacques et al., 2001; Refsum et al., 2006). Supplementation with these B vitamins has been shown to lower HcY concentrations (Eussen et al., 2006; McMahon et al., 2006; Smith et al., 2010; Clarke et al., 2014; Ford and Almeida, 2014). Moreover, data from the VITACOG study indicates that chronic supplementation with vitamins B_6_, B_12_ and folate is beneficial to cerebral structure, with reduced rates of total and regional cerebral atrophy due to significant reductions in HcY concentrations (Smith et al., 2010; Douaud et al., 2013; Jerneren et al., 2015). Lowering HcY through supplementation may also improve cognitive performance with several studies with older adults having observed improved memory performance and processing speed following HcY subsequent to B vitamin supplementation (Durga et al., 2007; de Jager et al., 2012). However, several systematic reviews and meta-analyses have indicated that lowering HcY through B vitamin supplementation does not improve cognitive function in older adults (Clarke et al., 2014; Ford and Almeida, 2014), though methodological weaknesses in these reviews has led to their results being criticized in more recent work (Smith and Refsum, 2016).

Overall, it would appear that lowering HcY through nutritional supplementation may benefit cerebral structural integrity in older adults. It may also be beneficial to cognitive function, though these latter effects remain controversial within the literature.

#### GPL supplementation and HcY concentrations

Choline is similar to B vitamins, in that its level of dietary intake appears to predict HcY concentrations in adults. A number of animal and plant based foods such as red meats, poultry, fish, eggs, and milk, but also broccoli, potatoes, green beans, legumes, and nuts, contain choline (Cho et al., 2006; Detopoulou et al., 2008). Following ingestion and absorption, choline may be oxidized into betaine, which functions as a methyl donor in the betaine-homocysteine-methyltransferase pathway, whereby HcY is converted to methionine in a reaction catalyzed by the enzyme betaine-homocysteine methyltransferase (Olthof et al., 2005). Importantly, increased ingestion or bioavailability of choline (or its metabolite betaine) has been observed to negatively predict HcY concentrations in adults (Schwab et al., 2002; Steenge et al., 2003; Cho et al., 2006; Chiuve et al., 2007; Atkinson et al., 2008; Detopoulou et al., 2008).

Choline is highlighted here as it forms the base attachment distinguishing PC (but also the sphingolipid known as sphingomyelin). In fact, choline represents 15% of PC molecular weight (Cheatham et al., 2012) thereby making PC an important carrier of choline that may be sourced from the diet. As such, greater ingestion of PC may be expected to negatively predict HcY concentrations. In several cross sectional studies utilizing FFQ data from the Framingham Heart Study (Cho et al., 2006) as well as the Nurses Health Studies (Chiuve et al., 2007), a number of dietary compounds containing choline were examined in relation to HcY. Cho et al. (2006), determined that estimated PC ingestion was inversely associated with HcY concentrations with similar associations being identified for other choline containing compounds including sphingomyelin, phosphocholine, and glycerophosphocholine. However, after controlling for additional factors related to HcY concentrations (e.g., B vitamin intake, caffeine, and alcohol consumption) PC was no longer associated with HcY concentrations, though other choline containing compounds remained as significant predictors. Interestingly, Chiuve et al. (2007) observed a negative association between choline from phosphocholine and glycerophosphocholine with HcY while there appeared to be a non-significant positive association with PC intake.

In perhaps the only study examining the direct effect of PC supplementation on HcY concentrations, Olthof et al. (2005) administered a daily oral dose of 34 g soybean lecithin (comprised of PC) delivering 2.6 g choline, to adult males aged 50–71 years over 2 weeks. Olthof et al. (2005) observed that HcY concentrations were 18% lower following treatment than placebo. However, it is important to note that the level of choline ingested in this study is well beyond the estimated mean daily intakes reported by both Cho et al. (2006) and Chiuve et al. (2007) (313 ± 61 and 323 mg/d, respectively). As such, it is possible that PC, due the provision of choline, will lower HcY at doses attainable through a combination of normal diet and supplementation than from diet alone.

Lowering HcY has been observed to benefit cerebral structural integrity in a number of clinical trials involving chronic B vitamin supplementation. As such, it is plausible that similar effects may be evident following chronic supplementation with PC due to the provision of choline. However, to date there has been minimal investigation of the use of PC as a means of lowering HcY. This is intriguing as choline, similar to select B vitamins, is an essential nutrient for the methylation of HcY. The relative lack of data determining the relationship between PC intake and HcY concentrations should be rectified by additional well-designed RCT's. The added utilization of advanced imaging methods in future studies will help determine how PC intake and subsequent HcY levels relate to cerebral structural integrity.

#### Lowering HcY and potential benefits to cerebral structure

Despite there being a paucity of work determining how PC supplementation benefits cerebral structure in aging humans, it is plausible that PC supplementation may benefit cerebral structure by lowering HcY and alleviating the detrimental effects theorized to be associated with elevated concentrations of HcY. One effect of elevated HcY is an increased risk of hyperphosphorylated tau buildup as well as an increased presence of neurofibrillary tangles (NFT) within cerebral tissues (Luo et al., 2007; Zhang et al., 2008; Popp et al., 2009; Wei et al., 2011; Hooshmand et al., 2013; Li et al., 2014). Both of these factors have been linked to poorer cerebral macro- and microstructural integrity in adults across the spectrum of cognitive function (Whitwell et al., 2008; Polvikoski et al., 2010; Tosun et al., 2010; Glodzik et al., 2011, 2012; Marnane et al., 2016; de Souza et al., 2017; Hoy et al., 2017; Kantarci et al., 2017).

Increased HcY concentrations appears to exacerbate the production of hyperphosphorylated tau through inhibition of kinases, such as protein phosphatase 2A (PP2A), which are responsible for tau dephosphorylation (Luo et al., 2007; Zhang et al., 2008; Wei et al., 2011). However, lowering HcY concentration (through B vitamin supplementation) has been observed to alleviate PP2A inactivation thereby lowering tau hyperphosphorylation (Zhang et al., 2008; Wei et al., 2011). Further, while there have been no trials examining HcY lowering and changes to NFT load, given that NFT are largely composed of hyperphosphorylated tau proteins, lowering HcY should theoretically influence the extent to which NFT are formed and therefore evident within cerebral tissues. Although data is limited, PC supplementation, due to the provision of choline, has been observed to lower HcY concentrations. As such, it is possible that by lowering HcY, PC supplementation (administered in moderate to high doses) may facilitate reduced concentrations of hyperphosphorylated tau and subsequently NFT, thereby supporting cerebral structural integrity in the long term. These effects remain to be investigated in older adults.

In addition to increased hyperphosphorylated tau and NFT load, elevated HcY may facilitate more subtle effects potentially leading to reduced cerebral structural integrity. Elevated HcY concentrations inhibit cellular PEMT activity leading to reduced production of PC containing ω3-PUFA thereby facilitating distorted concentration ratios of PC and its precursor PE within cellular membranes (Innis et al., 2003; Miller et al., 2003; Devlin et al., 2007; Selley, 2007).

Lowered production of PC containing DHA, due to PEMT inhibition, may also partly explain the reduced concentrations of DHA observed within biological tissues when HcY levels are elevated (Innis et al., 2003; Miller et al., 2003; Li et al., 2006; Devlin et al., 2007; Selley, 2007; Rasmussen et al., 2010; Huang et al., 2013; Kume et al., 2013; Iglesia et al., 2017). The production of PC containing ω3-PUFA such as DHA by the PEMT pathway is a key process for transporting DHA from the liver into plasma and onwards to cerebral tissue (Smith and Refsum, 2016). Ultimately, lower concentrations of ω3-PUFA such as DHA within membranes (including those within cerebral tissue) may then facilitate an increased risk of OxS and elevated inflammation, subsequently leading to poorer cardio- and cerebrovascular function (elevated blood pressure, arterial stiffness, and permeability of the blood-brain barrier). These factors will be discussed in later sections of this review.

PC is a major dietary source of choline. As such increased ingestion of PC via supplementation, likely facilitates increased choline bioavailability thereby lowering HcY concentrations and subsequently alleviating the inhibitory effects of HcY upon PEMT activity. This would influence the extent to which ω3-PUFA are present in cellular membranes (including those in cerebral tissue) due to restored *in vivo* synthesis of PC comprised of ω3-PUFA such as DHA. However, depending on the source from which it as derived, the administered PC may also contain ω3-PUFA. Supplementing with PC containing ω3-PUFA such as DHA would likely facilitate increased concentrations of these PUFA within cell membranes. This would impact the extent to which factors such as elevated OxS and inflammation are apparent, thereby decreasing the likelihood of disturbed cardio- and cerebrovascular function, and subsequently the extent to which cerebral structure deteriorates over time.

Although a great deal more work is required examining how PC ingestion relates to HcY concentrations, the limited data available indicates that PC supplementation, in high enough doses, may lower HcY concentrations. By lowering HcY, PC supplementation may benefit cerebral structural integrity and potentially cognitive function in a similar way to B vitamins. *It is hypothesized that chronic supplementation of GPL containing choline (i.e., PC) will significantly reduce HcY concentrations in older adults. Based upon earlier work whereby lowering HcY through nutritional supplementation benefited cerebral structural integrity, it is anticipated that HcY lowering via PC supplementation would likewise support cerebral structural integrity and subsequently cognitive functioning in older adults*.

### Oxidative stress

#### Risk of oxidative stress and cerebral structure in older adults

A ready supply of oxygen is essential for proper cellular functioning, although a consequence of oxygen metabolism is the formation of ROS (Chiurchiù et al., 2016). ROS are essential for many vital physiological functions including the destruction of pathogens during the inflammatory immune response and the regulation of smooth muscle tone (Popa-Wagner et al., 2013). Under normal conditions, ROS activity is balanced with that of endogenous antioxidants such as superoxide dismutase (SOD), glutathione peroxidase (GPx), and glutathione (GSH), but also dietary antioxidants (Chiurchiù et al., 2016). However, unbalanced ROS activity may result in oxidative damage to lipids and proteins comprising cellular membranes thereby impacting cellular membrane structure and functioning (Popa-Wagner et al., 2013; Von Bernhardi et al., 2015).

Damage to biological tissues due to unbalanced ROS activity is termed oxidative stress (OxS). Cerebral tissue may be particularly susceptible to OxS, due to it requiring a considerable amount of oxygen so as to sustain disproportionate metabolic activity. Other factors facilitating an increased risk of OxS within cerebral tissues include a marked production of ROS through neurochemical reactions as well as deposits of iron that may promote oxidation (Popa-Wagner et al., 2013).

An additional factor contributing to cerebral tissues susceptibility to OxS, is that the PUFA within cellular membranes are highly susceptible to oxidative damage by ROS (Chan et al., 1982; Brett and Rumsby, 1994; Bongarzone et al., 1995), with increased ROS activity (without a corresponding increase in antioxidant activity) having been observed to facilitate destruction of myelin lipids and proteins (Chia et al., 1983a,b; Konat and Wiggins, 1985; Bongarzone et al., 1995). However, there appears to be an absence of data linking ROS activity or measures reflecting OxS with cerebral structural integrity, as determined using magnetic resonance imaging (MRI), in older humans. It is highly likely that oxidation of lipids and proteins within cerebral tissue membranes, at least partly contributes to those reductions in cerebral microstructural integrity identified in cognitively normal (Davis et al., 2009; Burzynska et al., 2010; Westlye et al., 2010) but also cognitively impaired older adults (i.e., MCI or AD) (Bosch et al., 2012; Wang et al., 2012; Zhang et al., 2013). Though they are not perfect measures, the diffusion tensor imaging (DTI) metrics “radial diffusivity” and “axonal diffusivity” have been suggested to reflect myelin and axonal integrity, respectively, within cerebral white matter (Song et al., 2002, 2003; Sun et al., 2006). Future work utilizing these measures may provide additional insight into how ROS activity, and subsequently OxS, contributes to declining cerebral structural integrity with age.

Due to a heightened susceptibility of cerebral tissues to OxS, it follows that measures indicative of OxS, or an elevated susceptibility to OxS due to unbalanced ROS activity (e.g., poorer concentrations of endogenous or dietary antioxidants), may be predictive of poorer cognitive function. In fact, compared to healthy adults, those with MCI or AD appear to demonstrate reduced concentrations of endogenous and diet derived antioxidants (Rinaldi et al., 2003; Padurariu et al., 2010; Mandal et al., 2015). In addition, adults with MCI or AD also demonstrate elevated OxS than cognitively normal adults, evidenced by increased concentrations of malondialdehyde (MDA), F_2_ and F_4_ isoprostanes (measures of ω6 and ω3-PUFA oxidation, respectively), and protein carbonyls (Mangialasche et al., 2009; Padurariu et al., 2010; Torres et al., 2011).

Conversely, greater intake of dietary antioxidants (e.g., carotenoids) are associated with greater cognitive performance in older adults (Jama et al., 1996; Berr et al., 1998; Akbaraly et al., 2007; Wengreen et al., 2007), though conflicting data is available (Crichton et al., 2013). Moreover, higher concentrations of endogenous antioxidants, such as GPx and GSH appear to predict better performance on the mini-mental state examination as well as the clinical dementia rating scale but also improved executive functioning in older adults with MCI, AD or “cognitive dysfunction” (Umur et al., 2011; Mandal et al., 2015; Revel et al., 2015).

#### GPL supplementation and oxidative stress

Preclinical data appears to demonstrate that pre-treatment with GPL such as PS, may protect cells against the deleterious effects of elevated ROS, thereby reducing the risk of OxS. In one early study, Latorraca et al. (1993) suspended cultured human fibroblast cells in a solution containing acetaldehyde (37.5 mM) and 50 mU of xanthine-oxidase, which triggered a reaction resulting in high ROS production. Cell damage facilitated by increased ROS activity was quantified by measuring lactate dehydrogenase, with levels increasing by 40% when cells were exposed to both acetaldehyde and xanthine oxidase, compared to control (vehicle solution + acetaldehyde, but no xanthine oxidase). However, in cells pre-cultured with 13 μM PS for 4 days prior to suspension in the experimental solution, there was no significant increase in lactate dehydrogenase, suggesting that exposure to PS prevented tissue oxidative damage otherwise expected following increased ROS production.

Pre-culturing cells with GPL may facilitate decreased production of ROS, thereby lowering the risk of OxS occurring. Hashioka et al. (2007a) administered 400 ng/ml lipopolysaccharide (LPS) combined with 400 ng/ml of phorbol 12-myristate-12-acetate, to microglial cells sourced from rodents. This combination resulted in the production of the ROS and reactive nitrogen species—superoxide and nitric oxide, respectively, which together may form a powerful oxidant called peroxynitrite. However, when microglial cells were pre-cultured for 1 h with liposomes comprised of PS and PC or PC alone, both superoxide and nitric oxide production was markedly reduced. This would suggest that by lowering the production of superoxide and nitric oxide, treatment with liposomes comprised of PC and PS may facilitate reduced peroxynitrite production, lowering the likelihood of OxS. Similarly, Chaung et al. (2013) pre-cultured C6 cells (rodent glial cells modeling glioma) with 25 μM of DHA or 25 μM of PS, alone or in combination, for 24 h prior to these cells receiving electrical stimulation in order to stimulate ROS production. Across treatments, electrical stimulation of these glial cells successfully elevated ROS production, though for cells pre-cultured with either DHA or PS, ROS production was significantly lower. Moreover, the combination of DHA and PS appeared to facilitate even lower ROS production than when either treatment in isolation.

Prior work with animals indicates that supplementing with GPL containing ω3-PUFA may prevent OxS. Hiratsuka et al. (2008) fed rodents one of three experimental diets fortified with different lipid species. In the control diet lipids were issued as safflower oil (control), while experimental diets were fortified with the ω3-PUFA DHA delivered as either triglycerides or as GPL (PC, PE and SM) sourced from skipjack tuna ovaries. Rodents were fed their respective diets for a period of 5 weeks, though after the first 2 weeks' rodents received five daily injections of strepozotocin (40 mg/kg), so as to model diabetes, which subsequently caused elevated lipid peroxidation within cerebral tissue. Upon completion of the dietary intervention, no significant difference in cerebral lipid oxidation was identified between rodents within the control group or those receiving DHA as triglycerides. However, cerebral lipid oxidation was significantly lower in rodents fed DHA delivered as GPL, suggesting that supplementation with GPL containing DHA may lower the risk of OxS within cerebral tissue.

In addition to the above studies, others have determined that GPL supplementation may boost the concentrations of endogenous antioxidants. Liu et al. (2012) repeatedly administered pentylenetetrazol to induce epileptic-like seizures in rodents. This treatment facilitated a significant increase in the concentrations of reactive nitrogen species such as nitric oxide, while also reducing concentrations of endogenous antioxidants such as SOD within cerebral and liver tissues. Following seizure inducement, Liu et al. (2012) provided rodents with experimental diets fortified with 2.5 mg/kg DHA and/or 300 mg/kg PS for a period of 36 days. Dietary fortification with either DHA or PS was observed to increase SOD concentrations within cerebral tissue, whilst the combination of these nutrients boosted SOD concentrations within the liver. Nitric oxide was reduced in cerebral tissue following consumption of a diet fortified with DHA or PS, though liver concentrations were reduced following separate or combined treatment paradigms. Likewise, Zhang et al. (2015) observed that intracranial injection of 5.0 μL of amyloid-β_1−42_ facilitated a significant decline in SOD concentrations, while consumption of a diet enriched with PS sourced from bovine cortex (thereby containing DHA among other fatty acids) increased cerebral SOD expression in a dose dependent manner. More recently, Qu et al. (2016) modeled AD in rodents via intracerebral injection of amyloid-β_25−35_, subsequently reducing the concentrations of endogenous antioxidants such as SOD and GPx within the cortex and hippocampus. Compared to the AD control model, rodents modeling AD who received a medium (0.1 g/kg/d) or high (0.2 g/kg/d) dose of marine sourced PC (containing high levels of DHA and EPA) for 30 days demonstrated increased GPx within cortical tissue. Rodents that received a low (0.05 g/kg/d), medium or high dose of PC demonstrated elevated SOD activity within cortical tissue, although increased SOD activity within the hippocampus was only apparent following supplementation with the highest dose of PC. No treatment effects were observed within the hippocampus for GPx.

#### GPL supplementation and OxS—potential pathways of effect

There is data from a range of preclinical and clinical trials indicating that supplementation with GPL may reduce the risk of OxS within biological tissues, including cerebral tissue. GPL from marine sources (e.g., krill or squid), mammalian (e.g., cow or pig) brain as well as soybeans may reduce the risk of OxS through the provision of fatty acids, especially ω3-PUFA such as DHA or EPA. Although maintaining redox balance is complex and facilitated via a wide range of biological processes, discussion in this review will be limited to two potential pathways through which supplementation with GPL, due to the provision of ω3-PUFA, may modulate the activity or concentrations of ROS and endogenous antioxidants thereby lowering the risk of OxS.

The first pathway through which GPL containing ω3-PUFA may reduce the risk of OxS involves upregulation of transcription factors associated with the synthesis of endogenous antioxidants. Prior work indicates that when cells are incubated with ω3-PUFA (DHA, EPA, or ALA) there appears to be an increase in the concentrations of endogenous antioxidants such as SOD, GSH, and GPx (Di Nunzio et al., 2011, 2016; Saw et al., 2013). Increased concentrations of these endogenous antioxidants has been linked to elevated activity of NF-E2-related factor 2 (NrF2)—a factor involved in the transcription of genes coding for those antioxidants (Di Nunzio et al., 2011, 2016; Saw et al., 2013). These effects may occur in response to oxidation of ω3-PUFA such as DHA and EPA, with the byproducts resulting from oxidation of these lipids (e.g., 4-hydroxy-2E-hexenal) subsequently inhibiting factors known to decrease NrF2 nuclear translocation, such as Keap1 (Gao et al., 2007). These putative effects were examined recently by Zhang et al. (2014), who identified that the administration of fish oil (rodent models) or pretreatment with the ω3-PUFA's DHA and EPA (rodent embryonic neuronal cells *in vitro*) significantly reduced the destruction of neuronal cells following oxygen-glucose deprivation. Zhang et al. (2014) also observed that cells pretreated with DHA, exhibited greater nuclear translocation of NrF2 following oxygen glucose deprivation. Moreover, they observed that 4-hydroxy-2E-hexenal, an end product of ω3-PUFA oxidation, was a far more potent inducer of NrF2 activity than 4-hydroxy-2E-nonenal—an end product of ω6-PUFA oxidation.

As indicated earlier, supplementation with ω3-PUFA is efficacious for elevating the bioavailability of these PUFA within biological tissues (Popp-Snijders et al., 1986; Bourre et al., 1993; Yaqoob et al., 2000; Bourre and Dumont, 2002; Favrelière et al., 2003; Kew et al., 2003, 2004; Browning et al., 2012). However, delivery of ω3-PUFA as PL may be a more effective for elevating tissue concentrations of these lipids than triglycerides (Ramprasath et al., 2013). Typically, increased concentrations of ω3-PUFA within membranes occurs at the expense of ω6-PUFA such as ARA (Calder, 2015). As such, increasing the bioavailability of ω3-PUFA within biological tissues, may increase the likelihood of these species of PUFA being oxidized thereby increasing the production of 4-hydroxy-2E-hexenal, rather than 4-hydroxy-2E-nonenal (from ω6-PUFA) leading to increased NrF2 activity and production of endogenous antioxidants. This process may account for the increased concentrations of endogenous antioxidants and therefore reduced risk of OxS, following GPL administration in earlier studies.

A second pathway through which GPL containing ω3-PUFA may reduce the risk of OxS is by downregulating the activity of select enzymes that produce ROS. One particular class of enzymes associated with ROS production are the nicotinamide adenine dinucleotide phosphate (NADPH) oxidases, abbreviated as Nox (Lambeth, 2004; Bedard and Krause, 2007). Nox are membrane bound proteins which facilitate electron transfer from NADPH onto molecular O_2_, thereby generating ROS, particularly superoxide. Dysregulated Nox activity may contribute to endothelial dysfunction, distorted smooth muscle growth as well as inflammation (Giordano and Visioli, 2014), and has been linked to an increased risk of MCI and AD, while also predicting Braak stage—a measure of AD pathology (Bruce-Keller et al., 2010; Ansari and Scheff, 2011). There are a number of different Nox enzymes (e.g., Nox1, Nox 2, Nox 3, Nox 4, Nox 5) which may be expressed in low to high concentrations within a wide variety of biological tissues (Lambeth, 2004; Bedard and Krause, 2007). Importantly, the activity of Nox proteins is regulated by a number of different proteins including p22^phox^, p47^phox^, p67^phox^, p40^phox^, and the GTPase Rac (Bedard and Krause, 2007).

Supplementation with GPL may downregulate Nox activity, subsequently lowering the risk of OxS, assuming the GPL contain ω3-PUFA. Richard et al. (2009) pre-cultured human aortic endothelial cells with DHA for 48 h, observing that DHA treatment facilitated a reduction in Nox4 expression and subsequently ROS production, which was otherwise upregulated due to exposure to angiotensin II and IL-1β. Additionally, the topical application of DHA to hairless mouse skin significantly attenuated the expression of Nox4 following exposure to ultraviolet radiation in a dose dependent manner compared to control (Rahman et al., 2011). In a more recent study by Depner et al. (2013), rodents were fed chow with a nutritional composition resembling that of the western diet regularly consumed by humans. Some of the rodents also received supplementation with ω3-PUFA's such as EPA and/or DHA, whereas controls only received olive oil. Although Depner et al. (2013) did not observe increased NrF2 expression following ingestion of any diet with added supplementation of ω3-PUFA, they did observe marked reduction in Nox regulating proteins including p22^phox^, p40^phox^, p47^phox^, p67^phox^, as well as RAC1, a member of the RAC subgroup of GTPase. Based upon the findings of these earlier studies, it is possible that supplementation of GPL (containing ω3-PUFA) may reduce the risk of OxS by down regulating the activity of enzymes known to produce ROS, as well as factors that stimulate the activity of those enzymes.

Overall, it would appear that supplementation with GPL, particularly those also containing ω3-PUFA, may modify the activity of several different protein messengers leading to reduced production of ROS, but increased synthesis of endogenous antioxidants, thereby lowering the risk of OxS. *As such, it is anticipated that chronic supplementation with GPL (containing* ω*3-PUFA*) *is expected to facilitate a greater balance between the concentrations of ROS and endogenous antioxidants in older adults. This will likely manifest as lower concentrations of markers indicative of OxS. It is anticipated that in an aging sample, lower expression of OxS related markers or elevated expression of anti-oxidants (endogenous and dietary) will be associated with greater cerebral structural integrity, which in turn would be predictive of greater cognitive functioning*.

### Inflammation

#### Inflammation and cerebral structure in older adults

Inflammation is an integral component of the immune response following injury to body tissues or infection. An important regulating influence of the inflammatory response is the production and release of cellular messengers specialized for either promoting or alleviating inflammation. Common pro-inflammatory messengers include interleukin (IL)-1β, IL-6, IL-8, IL-12, tumor necrosis factor alpha (TNF-α), and C-reactive protein (CRP), while examples of anti-inflammatory messengers include IL-4 and IL-10 (Baune et al., 2008; Marioni et al., 2010).

With increasing age, there appears to be a shift toward an immunologically primed state. This has been identified in cerebral tissue, such as the hippocampus, where the inflammatory response is typically of a greater magnitude and duration in older adults (for a review see Barrientos et al., 2015). This is an important observation in the context of neurocognitive health as data from multiple studies indicate that elevated concentrations of pro-inflammatory messengers, such as those listed earlier, are predictive of poorer cerebral structural integrity. These detrimental effects are evident as lower regional cerebral tissue volumes, increased volume of white matter lesions, as well as poorer cerebral microstructure (Baune et al., 2009; Wersching et al., 2010; Nagai et al., 2011; Bettcher et al., 2012, 2014, 2015; Miralbell et al., 2012; Satizabal et al., 2012; Arfanakis et al., 2013; Taki et al., 2013b; Sudheimer et al., 2014; Jiang et al., 2015).

Likewise, there appears to be a link between higher concentrations of pro-inflammatory messengers and poorer cognitive performance or increased rate of cognitive decline with age (Yaffe et al., 2003; Dimopoulos et al., 2006; Wright et al., 2006; Marioni et al., 2010; Economos et al., 2013; Mooijaart et al., 2013; Adriaensen et al., 2014; Tegeler et al., 2016). Greater concentration of pro-inflammatory messengers are evident in adults diagnosed with MCI or dementia compared to their cognitively normal counterparts (Dimopoulos et al., 2006; Magaki et al., 2007; Bermejo et al., 2008; Troller et al., 2010), with others having identified an increased risk of dementia in conjunction with elevated inflammation (Schmidt et al., 2002; Engelhart et al., 2004).

Interestingly, some studies appear to demonstrate a significant reduction in the strength of the relationship between inflammation and cognition after controlling for cerebral structural integrity (Arfanakis et al., 2013; Marsland et al., 2015). These results suggest that inflammation may detrimentally influence cognitive function, in part by promoting the deterioration of cerebral tissues. As such, the identification of interventions efficacious for modulating inflammatory messengers may be paramount for supporting cerebral structure, and subsequently cognitive function, in older adults.

#### GPL supplementation and inflammation

Data from earlier pre-clinical studies demonstrate that GPL supplementation may modify the concentrations of pro-inflammatory messengers. In several studies, Treede et al. (2007, 2009) observed that administering TNF-α to caco-2 cells (human intestinal epithelial cells) triggered the activation of nuclear factor kappa-B (NF-KB), an important factor associated with the transcription of genes coding for pro-inflammatory messengers and the subsequent upregulation of these signaling messengers. However, simultaneous treatment with PC prevented NF-KB activation and subsequently lower concentrations of pro-inflammatory messengers (such as additional TNF-α) relative to control cultures. Similarly, cultures of human microglial cells exposed to with liposomes comprising PS and PC (sourced from pig brain or egg yolks, respectively), prior to the application of amyloid-β and interferon-γ, demonstrated lower concentrations of TNF-α (Hashioka et al., 2007b).

Building on the pre-clinical literature, Hartmann et al. (2009) use a rodent model of arthritis (involving injection of a solution containing carrageenan and kaolin into the knee joint). The injection of this solution triggered an inflammatory response consistent with arthritis—specifically increased heat, swelling and pressure sensitivity, though only within the joints that received the injection. Importantly, these effects were abated via oral administration of a non-steroidal anti-inflammatory drug or PC (dose of 150 mg/Kg)—though only the latter decreased the number of adherent leukocytes in the affected joints. In another study, increased concentrations of TNF-α and IL-6 were elicited in rodents following injection of LPS. However, rodents fed an experimental diet fortified with PC (1%) demonstrated significantly lower concentrations of TNF-α following LPS injection when compared to control rodents (Tokes et al., 2011). Similarly, consumption of a diet fortified with soybean derived PC (1%) facilitated anti-inflammatory effects in the gastrointestinal tract of dogs following small intestine ischemia (Ghyczy et al., 2008). In other work, the concentrations of both TNF-α and IL-6 were reduced in rodents which received PC supplementation for 3 days prior to, and 5 days after, an enema with trinitrobenzenesulfonic acid (Kovacs et al., 2012). Further, Jung et al. (2013) modeled multiple organ injury via LPS injection in otherwise healthy rodents. LPS injection triggered an increase in pro-inflammatory messengers such as TNF-α and IL-6, as well as the anti-inflammatory messenger IL-10. However, in rodents administered PC, a significant reduction in both TNF-α and IL-6 was apparent, while concentrations of IL-10 were unaffected.

Although the weight of research examining the concentration of inflammatory messengers in response to GPL focuses on PC, and to a lesser extent PS, several studies have also investigated PE. Despite observing anti-inflammatory effects in response to PC supplementation, Treede et al. (2007, 2009) were unable to replicate these effects when supplementing with PE. In a later study, Eros et al. (2009) modeled pleurisy (inflammation of lung tissue and tissue lining the chest cavity) in rodents via injection of 100 μL saline containing 2% carrageenan into the thoracic cavity at the 6th intercostal space. Carrageenan injection triggered an increase in total leukocyte count and as well as elevated leukocyte accumulation within lung tissue. Importantly, inflammation was reduced in rodents fed a special diet containing PC, PE and N-acylphosphatidylethanolamine or NAPE (diet composition-−1% PC, 0.4% PE, and 0.1% NAPE) for the 7 days prior to injection of carrageenan and 48 h thereafter. However, it is difficult to disentangle the anti-inflammatory effects associated with PC and those potentially from either PE or NAPE, as only combined supplementation was investigated.

#### GPL supplementation and inflammation—potential pathways of effect

The available literature suggests that PC supplementation may be capable of beneficially modifying inflammation. These effects are potentially due to PC being a significant source of choline (Cho et al., 2006; Chiuve et al., 2007) and therefore stimulating the cholinergic anti-inflammatory pathway. In this pathway, efferent fibers of the vagus nerve interact with acetylcholine receptors, notably α7 nicotinic acetylcholine (α7nACh) receptors on macrophages and other non-neural cytokine producing cells. The release of acetylcholine from vagus nerve activates these receptors which in turn facilitates inhibitory effects upon NF-KB, resulting in downregulated transcription of genes associated with pro-inflammatory messengers (Gallowitsch-Puerta and Pavlov, 2007).

In several studies, choline has been observed to function as an α7nACh receptor agonist. Parrish et al. (2008) cultured rodent macrophage cells with LPS in the presence or absence of choline for 4 h prior to measuring TNF-α concentrations. Choline treatment appeared to lower the concentrations of TNF-α following the presentation of LPS in a dose-dependent manner. This decline in TNF-α was found to have occurred because of reduced NF-KB activity. Parrish et al. then proceeded to demonstrate these effects in rodents, through intraperitoneal injection of choline (5 or 50 mg/kg) or saline at two time points (6 h as well as 30 min) prior to an injection of endotoxin (6 mg/kg). The higher choline dose reduced TNF-α concentrations in rodents previously injected with endotoxin, due to reduced NF-KB activity following activation of α7nACh receptors. Importantly, these results were then replicated in human whole blood and cultured macrophages. Similar observations were also made by Gurun et al. (2009) following administrations of CDP-choline (precursor to PC, but also containing choline) in rodents via intraplantar injection. These authors also observed that the anti-inflammatory effects of choline occurred in response to choline stimulating α7nACh receptors and subsequently suppressing NF-KB activity and therefore production of pro-inflammatory messengers. Though more work is needed to understand this potential mechanism, it is possible that PC, as a significant source of choline may influence the concentrations of pro-inflammatory messengers through the cholinergic anti-inflammatory pathway. This likely accounts for the empirical observations discussed earlier, whereby PC administration was associated with reduced concentrations of pro-inflammatory messengers such as TNF-α (Treede et al., 2007, 2009).

An additional pathway through which GPL supplementation may influence inflammation is through modifying the fatty acid composition of cell membranes, including inflammatory cells. Previous work has indicated that supplementation with ω3-PUFA primarily within PL facilitates increased bioavailability of ω3-PUFA within serum and membrane bound PL (Ramprasath et al., 2013). Increased ω3-PUFA within membrane GPL typically occurs at the expense of ω6-PUFA such as ARA (Calder, 2015). As such, there may be a proclivity toward the release of ω3-PUFA such as EPA or DHA, rather than the ω6-PUFA ARA, through the action of phospholipase A_2_, during the inflammatory response. Once released, these fatty acids may be metabolized by cyclooxygenase (COX) and/or lipoxygenase (LOX) enzymes resulting in the production of eicosanoids. Eicosanoids resulting from ARA metabolism are pro-inflammatory as they stimulate the production of inflammation inducing messengers such as TNF-α, IL-6, IL-8, and IL-1β (Wall et al., 2010; Serhan and Petasis, 2011). However, eicosanoids produced through metabolism of ω3-PUFA, particularly EPA, exert far less potent pro-inflammatory effects than those associated with ARA (Wall et al., 2010; Calder, 2015). Moreover, the metabolism of EPA or DHA by COX and LOX enzymes facilitates the synthesis of powerful anti-inflammatory and inflammation resolving agents such as resolvins, maresins and protectins (Bannenberg and Serhan, 2010; Serhan and Petasis, 2011; Calder, 2015). Furthermore, increased bioavailability of ω3-PUFA may help facilitate reduced nuclear translocation of NF-KB and therefore the transcription of genes associated with pro-inflammatory messengers (Wall et al., 2010; Calder, 2015).

*Overall, it is anticipated that supplementation with GPL, especially those delivering choline and/or* ω*3-PUFA such as DHA, will beneficially modify the concentrations of inflammatory messengers. As chronic exposure to elevated concentrations of pro-inflammatory messengers appears to predict poorer cerebral structural integrity in older adults, it is predicted that lowering exposure to pro-inflammatory messengers will benefit cerebral structure. Subsequently, we would expect these effects to further benefit cognitive function in older adults*.

### Cardiovascular and cerebrovascular function

#### Cardio/cerebrovascular function and cerebral structure in older adults

Cardio- and cerebrovascular function are also pertinent factors predicting cerebral structure in older adults. One common measure of cardiovascular function is blood pressure (BP), specifically systolic and diastolic pressures (SBP or DBP, respectively). In middle aged and older adults, elevated BP (systolic and/or diastolic) or hypertension (clinically diagnosed high SBP and DBP) have been observed to predict poorer cerebral macrostructural integrity reflected as reduced total and regional cerebral volume (Den Heijer et al., 2003a, 2005; Raz et al., 2003; Beauchet et al., 2013), cortical thinning (Leritz et al., 2011; Alosco et al., 2014; Gonzalez et al., 2015), but also greater severity of cerebral white matter lesions or hyperintensities (Goldstein et al., 1998; Guo et al., 2009; Allan et al., 2015). Elevated BP also appears to predict poorer cerebral microstructural integrity (Kennedy and Raz, 2009; Leritz et al., 2010; Salat et al., 2012). An additional measure of cardiovascular function is arterial stiffness (AS). Increased AS has been observed to predict lower cerebral volume (Tsao et al., 2013; Lilamand et al., 2016) as well as increased severity of cerebral white matter lesions or hyperintensities (Henskens et al., 2008; Mitchell et al., 2011; Singer et al., 2014; van Sloten et al., 2015; Tsao et al., 2016). Both elevated BP and AS may contribute to poorer cerebral structural integrity, in part, due to chronically elevated pulsatile pressures damaging cerebral microvasculature. This may in turn facilitate reduced cerebral perfusion leading to ischemia and subsequent cerebral tissue decay (Gonzalez et al., 2015; van Sloten et al., 2015; Lilamand et al., 2016).

With an apparent association between poorer cardiovascular function and cerebral structural integrity, it follows that poorer cardiovascular function is predictive of reduced cognitive function. Elevated BP has been observed to predict poorer cognitive performance as well as an increased risk of developing MCI or dementia (Launer et al., 2000; Whitmer et al., 2005; Köhler et al., 2014; Chen et al., 2015; Alipour and Goldust, 2016) with similar effects having been observed in adults with elevated AS (Singer et al., 2014; Hajjar et al., 2016; Lim et al., 2016; Pase et al., 2016; Meyer et al., 2017). Given that there is a preponderance for increased BP (Neaton and Wentworth, 1992; Lloyd-Jones et al., 2005) and AS (Benetos et al., 2002; Mitchell et al., 2004) with age, targeting these outcomes with nutritional interventions may be benefit cerebral structural integrity, and therefore cognitive function in older adults.

Similarly, “blood-brain barrier” (BBB) integrity/permeability (an important measure of cerebrovascular function), may also influence cerebral structural integrity in older adults. The BBB is comprised of tightly compacted endothelial cells of capillaries perfusing cerebral tissue. Gases such as O^2^ and CO^2^ may freely diffuse across these capillary endothelial cells along their concentration gradients. However, the presence of “tight junctions,” composed of transmembrane proteins such as occluding and claudins, but also junction adhesion molecules, limits the paracellular flow of water, ions and other large molecules. Larger molecules may cross the BBB (either entering or leaving cerebral tissue), though movement of such molecules is normally dependent upon complex receptor and transporter systems (Popescu et al., 2009; Zeevi et al., 2010).

Maintenance of BBB integrity and therefore selective permeability is essential for ensuring optimal health of cerebral tissues. However, there appears to be a tendency toward reduced BBB integrity and increased permeability with older age. Pelegri et al. (2007) and Del Valle et al. (2009) have both observed increased BBB permeability with age in a rodent model of accelerated senescence (SAMP8). Likewise, reduced BBB permeability was identified in older relative to younger human adults in a systematic review and meta-analysis published around the same time (Farrall and Wardlaw, 2009).

Changes in BBB permeability with age appears to be predictive of the integrity of cerebral tissue, but also cognitive function, in older adults. In their systematic review, Farrall and Wardlaw (2009) identified a number of studies showing that elevated BBB permeability was correlated with increased severity of cerebral white matter lesions. Likewise, elevated BBB permeability is predictive of poorer memory (Wang et al., 2006) and performance on the mini-mental state examination (van de Haar et al., 2016). There also appears to be a preponderance for elevated BBB permeability in adults with either dementia (Alzheimer's and vascular subtypes) or MCI, relative to cognitively normal adults (Farrall and Wardlaw, 2009), particularly within the hippocampus, but also cerebral cortex and deep gray matter (Wang et al., 2006; Montagne et al., 2015; van de Haar et al., 2016). It is possible that increased BBB permeability detrimentally influences cognitive function through deleterious effects upon cerebral structure. Further work utilizing advanced structural neuroimaging methods, computerized tasks sensitive to subtle cognitive change, and importantly larger (and cognitively diverse) participant samples, will provide further insight into how changes to BBB permeability influence cerebral structural integrity and cognitive function with age.

#### GPL supplementation and cardio/cerebrovascular function

As indicated earlier, GPL derived from marine sources (e.g., krill), but also mammalian brain or soybean may function as a source of ω3-PUFA. There is evidence suggesting that increased ω3-PUFA intake, or bioavailability, is beneficial to BP (Mori et al., 1999; Paschos et al., 2007; Takeuchi et al., 2007; Theobald et al., 2007; Witte et al., 2013) though such effects may be limited to adults with untreated (or poorly treated) hypertension (Campbell et al., 2012; Miller et al., 2014; Minihane et al., 2016). Similar benefits may also be observed for AS. In a systematic review and meta-analysis, Pase et al. (2011) identified that supplementation with ω3-PUFA was efficacious for improving pulse wave velocity and arterial compliance, indicating reduced AS. In a later trial, Pase et al. (2015) observed that daily supplementation of 6 g fish oil (480 mg DHA + 480 mg EPA) for 16 weeks significantly lowered central AS as measured by aortic augmentation pressure. Additional support for an association between ω3-PUFA intake/bioavailability and levels of arterial stiffening is provided by several studies with middle aged and older adults whereby greater tissue concentrations of ω3-PUFA predicted lower AS (Anderson et al., 2009; Sekikawa et al., 2013; Reinders et al., 2015; Lee et al., 2016). Given that prior work has indicated that GPL supplementation may effectively elevate the concentrations of ω3-PUFA within biological tissues (Ramprasath et al., 2013), it is anticipated that supplementation with GPL containing ω3-PUFA would also benefit measures of cardiovascular function such as BP or AS.

To date, very few studies have investigated whether GPL supplementation influences cardiovascular function in older humans. In one study, Vakhapova et al. (2011) administered a daily dose of PS (300 mg PS and 79 mg DHA and EPA; ratio of 3:1) to 157 older adults (mean age was ~72 years) daily for a period of 15 weeks. Following an initial 15-week double blind treatment phase, no differences were evident between treatment or control groups for either SBP or DBP. However, the trial was continued for an additional 15-weeks in an open label extension, though with a reduced dose of PS (100 mg PS and 26 mg DHA and EPA). The extension included 121 adults from the original trial phase, separated into two groups. Adults who previously received the PS supplement were designated as “continuers” whereas those originally receiving the placebo were termed “naïve.” Following an additional 15 weeks' intervention, Vakhapova et al. (2011) identified that in the adults designated as “continuers” DBP was significantly reduced from baseline, whereas no change was identified in the “naïve” group. However, it cannot be determined whether these effects are attributable to PS or the additional ω3-PUFA (or both), as simultaneous administration was the only experimental treatment. In another open label trial, Richter et al. (2013) administered a daily dose of 300 mg PS derived from soybeans (thereby containing ALA, which may be converted to longer chain ω3-PUFA such as DHA) to 30 adults aged between 50 and 90 years, for a period of 12 weeks. Following treatment, Richter et al. (2013) identified significant reductions to both SBP and DBP.

Although beneficial effects to cardiovascular function have been observed following GPL supplementation, contrary data are available. Jorissen et al. (2002) reported data relating to the safety of soybean derived PS when administered to older adults with AAMI in doses of either 300 or 600 mg, for a period of 12 weeks. The authors report that there was an absence of any significant group differences in any BP measure upon completion of the supplementation period. However, the absence of beneficial effects may be due to important methodological factors. In an earlier publication using data from the same study, Jorissen et al. (2001) suggested that a lack of cognitive benefits following GPL supplementation may be due to their PS having degraded by 50% by 15 months' post production. Although Jorissen et al. (2001) report that PS administration ended ~11 months after the treatments were manufactured, degradation may still have been advanced enough to minimize the likelihood of cardiovascular effects. Similar to Jorissen et al. (2002), Richter et al. (2013) administered PS in the form of gelatin capsules, though Richter et al. (2013) report that their capsules were specially engineered in order to GPL stability. Another pertinent difference between these studies is their use of parallel treatment groups. Jorissen et al. (2001, 2002) utilized a double blind, parallel groups design. Vakhapova et al. (2011) appears to have only performed within groups comparisons to determine whether adults designated “continuers” or “naïve” experienced cardiovascular benefits with no additional analyses having been performed so as to identify potential between groups effects. Likewise, Richter et al. (2013) performed an open label trial with no placebo group, so changes to cardiovascular function were only determined through within groups analyses. Lastly, there were marker differences in the samples sizes between these studies. Jorissen et al. (2002) and Vakhapova et al. (2011) included approximately 130 participants each, whereas Richter et al. (2013) only included 30 (26 by end of study). These differences in trial design, and size, may begin to account for the differences in results between these studies. As such, while GPL supplementation may benefit cardiovascular functioning, additional well designed clinical trials are required to confirm these effects in older adults.

While there has been some investigation of cardiovascular benefits following GPL supplementation, there appears to have been no clinical trials do date examining how GPL supplementation influences cerebrovascular function, particularly BBB permeability. Despite this, it is plausible that benefits to BBB permeability in older adults will be apparent following chronic ingestion of GPL containing choline and/or ω3-FA. Potential mechanisms through which GPL supplementation may benefit BBB permeability are highlighted in the following section.

#### GPL supplementation and cardio/cerebrovascular function-potential pathways of effect

Although multiple factors likely contribute to poorer cardio and cerebrovascular function, it has been suggested that both OxS and inflammation may be particularly important. Increased ROS activity, as well as OxS, are suggested to be key factors in the etiology of hypertension (Vaziri and Rodríguez-Iturbe, 2006; Briones and Touyz, 2010; Wu and Harrison, 2014). In addition, elevated inflammation is suggested to predict increased BP or the presence hypertension (Vaziri and Rodríguez-Iturbe, 2006; Dinh et al., 2014), though it may do so via reciprocal relationships with both OxS and endothelial dysfunction (Dinh et al., 2014). Furthermore, OxS has been positively linked with elevated AS (Patel et al., 2011; Kawamoto et al., 2016) potentially via remodeling of the vascular wall (Fleenor, 2013). Likewise, chronically elevated inflammation may initiate detrimental changes to the structural makeup of arterial walls, ultimately contributing to elevated AS (Jain et al., 2014).

Likewise, unbalanced ROS activity leading to OxS, but also elevated concentrations of certain pro-inflammatory messengers are suggested to facilitate increased BBB permeability (for reviews see Pun et al., 2009; Oakley and Tharakan, 2014; Rochfort and Cummins, 2015). Among other mechanisms, detrimental changes to tight junction proteins are often highlighted as a cause of increased BBB permeability following OxS or elevated concentrations of pro-inflammatory messengers such as TNF-α or IL-6 (Pun et al., 2009; Coisne and Engelhardt, 2011; Elahy et al., 2015; Varatharaj and Galea, 2017). Elevated HcY also predicts elevated BBB permeability in rodents (Kamath et al., 2006; Lominadze et al., 2006; Beard et al., 2011; Rhodehouse et al., 2013). Reduced BBB permeability has also been observed alongside elevated HcY in adults with MCI (Lehmann et al., 2003). Each of these risk mechanisms has been shown to be modifiable through supplementation with GPL containing choline and/or ω3-PUFA (see previous sections). Subsequently, it is anticipated that GPL supplementation, would further benefit BBB permeability in older adults. This remains to be investigated in well-designed clinical trials.

Although much more work is required, there is both direct and indirect evidence to suggest that GPL supplementation may benefit measures associated with cardio- and cerebro-vascular function in older adults. *As such, it is hypothesized that chronic supplementation with GPL containing choline and/or* ω*3-PUFA will improve the aforementioned measures of cardio- and cerebro-vascular function in older adults, thereby supporting cerebral structural integrity and cognitive functioning in older adults*.

### Summary

**Figure 3:**
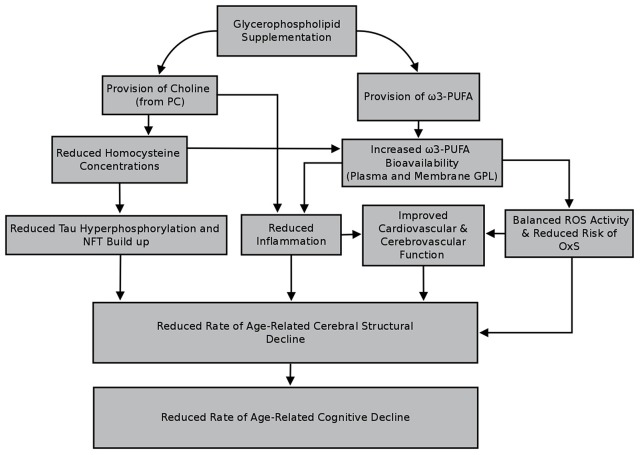
Select pathways through which glycerophospholipid supplementation may benefit cerebral structure and cognitive function in older adults.

Chronic supplementation with GPL has been observed to benefit cognitive function in animals and older adults (see introduction, as well as Tables 1–3). There is also data supporting the notion that GPL supplementation is beneficial to cerebral structure, though to date these effects have only been examined for in rodents. Despite a paucity of work in older humans, it is plausible that beneficial effects to cerebral structure are achievable, especially if the GPL administered contains choline and/or ω3-PUFA (DHA, EPA, or ALA). Long-term supplementation of GPL containing the aforementioned nutrients is expected to elevate the bioavailability of these nutrients, thereby resulting in modification of, or protection against, a number of factors associated with the destruction of cerebral tissues (see sections Homocysteine, Oxidative Stress, Inflammation, and Cardiovascular and Cerebrovascular Function). By minimizing or improving the factors outlined in this review, it is possible that GPL supplementation will lower the rate of cerebral structural decline over time. There is also the possibility that supplementation may facilitate at least mild repair to cerebral tissue given that GPL are major components of cellular membranes. Whether it be by reduced rate of decline, repair, or a combination of both, it is anticipated that by supporting cerebral structure, GPL supplementation may also influence the trajectory of cognitive decline with increasing age. Figure 3 outlines select pathways through which GPL supplementation may benefit cerebral structure, and subsequently cognitive function, in older adults. These effects should be examined in future well-designed clinical trials incorporating the use of advanced structural neuroimaging methods.

## Conclusion

There is substantial research indicating that cerebral structural integrity, at both the macro- and microstructural levels, is reduced with age. Modifying nutritional intake is quickly becoming recognized as a means of supporting cerebral structure with age, with a number of trials indicating that chronic supplementation with B vitamins, ω3-PUFA, or resveratrol, mediates reduced cerebral deterioration over time, perhaps even facilitating repair. This review discusses a number of different pathways through which benefits to cerebral structure may occur in response to GPL supplementation, thereby providing a theoretical basis for future human clinical trials.

Given that cerebral macro- and microstructural integrity is a pertinent predictor of cognitive function in older adults, it is plausible that through supporting cerebral structure, a reduced rate of cognitive deterioration may become apparent. Improving the trajectory of age-related cerebral deterioration and therefore cognitive decline through readily accessible interventions such as nutritional supplementation, may help lower the risk, and delay the onset, of age related conditions such as AAMI and MCI. Moreover, it may be possible to delay the onset of pathological conditions such as dementia, thereby contributing to a reduced incidence of this disease (Brookmeyer et al., 2007). Given the ease at which nutrition can be modified, and the relative absence of harmful side effects, nutritional supplementation, particularly with GPL, may well be a useful intervention for supporting neurocognitive health with increasing age.

## Author contributions

JMR: Drafted the manuscript, with all additional authors (DJW, HM, AS, and AP) making significant contributions to the overall planning and development of the manuscript.

### Conflict of interest statement

JMR is the recipient of an Australian Government Postgraduate research scholarship. DJW has received research funding and consultancy fees from Abbott Nutrition, Bayer Healthcare, and Neurobrands. HM has received research funding from Swisse-Wellness. AS has received research funding and consultancy fee from Abbott Nutrition, Australian Wine Research Institute, Barilla, Bayer Healthcare, Blackmores, Cognis, Cyvex, Dairy Health Innovation Consortium, Danone, Ginsana, GlaxoSmithKline Healthcare, Masterfoods, Martek, Naturex, Nestlé, Novartis, Red Bull, Sanofi, Unilever, Verdure Sciences, Wrigley. AP has received research funding and consultancy fees from Biostime, Blackmores, DSM, LifeVantage, Novasel Australia, Enzo Nutraceuticals and Swisse Wellness. AP was previously a member of the Scientific Advisory Panel for Swisse Wellness. Both DJW and AS have received grant funding from ARLA Foods to perform a clinical trial investigating the effects of a phospholipid supplement on neurocognitive health in older adults. AS has received research funding, consultancy and honoraria from the food industry.

## Abbreviations

AAMI: Age-associated memory impairment
MCI: Mild cognitive impairment
AD: Alzheimer's dementia
PL: Phospholipid
GPL: Glycerophospholipid
PC: Phosphatidylcholine
PE: Phosphatidylethanolamine
PS: Phosphatidylserine
CDP-choline: Cytidine disphosphocholine
PEMT: Phosphatidylethanolamine *N*-methyltransferase
PSS1: Phosphatidylserine synthase-1
PSS2: Phosphatidylserine synthase-2
PSD: Phosphatidylserine decarboxylase
PUFA: Polyunsaturated fatty acid
ω3/ω6: Omega 3 or 6
EPA: Eicosapentaenoic acid
DHA: Docosahexaenoic acid
ALA: α-Linolenic acid
AA: Arachidonic acid
HcY: Homocysteine
NFT: Neurofibrillary tangles
OxS: Oxidative stress
ROS: Reactive oxygen species
SOD: Superoxide dismutase
GSH: Glutathione
GPx: Glutathione peroxidase
MDA: Malondialdehyde
NrF2: NF-E2 related factor 2
NADPH: Nicotinamide adenine dinucleotide phosphate
Nox: NADPH oxidase
IL: Interleukin
TNF-α: Tumor necrosis factor alpha
NF-kB: Nuclear factor kappa B
COX: Cyclooxygenase
LOX: Lipoxygenase
BP: Blood pressure
SBP: Systolic blood pressure
DBP: Diastolic blood pressure
AS: Arterial stiffness
BBB: Blood-brain barrier.
